# Harmonization and standardization of data for a pan-European cohort on SARS- CoV-2 pandemic

**DOI:** 10.1038/s41746-022-00620-x

**Published:** 2022-06-14

**Authors:** Eugenia Rinaldi, Caroline Stellmach, Naveen Moses Raj Rajkumar, Natascia Caroccia, Chiara Dellacasa, Maddalena Giannella, Mariana Guedes, Massimo Mirandola, Gabriella Scipione, Evelina Tacconelli, Sylvia Thun

**Affiliations:** 1 Berlin Institute of Health (BIH), Charité – Universitätsmedizin Berlin, Berlin, Germany; 2 University of Bologna, Bologna, Italy; 3 Cineca Consorzio Interuniversitario, Bologna, Italy; 4 University of Verona, Verona, Italy

**Keywords:** Medical research, Epidemiology

## Abstract

The European project ORCHESTRA intends to create a new pan-European cohort to rapidly advance the knowledge of the effects and treatment of COVID-19. Establishing processes that facilitate the merging of heterogeneous clusters of retrospective data was an essential challenge. In addition, data from new ORCHESTRA prospective studies have to be compatible with earlier collected information to be efficiently combined. In this article, we describe how we utilized and contributed to existing standard terminologies to create consistent semantic representation of over 2500 COVID-19-related variables taken from three ORCHESTRA studies. The goal is to enable the semantic interoperability of data within the existing project studies and to create a common basis of standardized elements available for the design of new COVID-19 studies. We also identified 743 variables that were commonly used in two of the three prospective ORCHESTRA studies and can therefore be directly combined for analysis purposes. Additionally, we actively contributed to global interoperability by submitting new concept requests to the terminology Standards Development Organizations.

## Introduction

The multinational initiative ORCHESTRA, funded by the European Commission, aims at establishing a new European-wide cohort. Based on existing and new large-scale clinical studies with different population cohorts, data from several centers and countries will be integrated to advance research on COVID-19. The work presented here shows how semantic interoperability was established within three studies belonging to ORCHESTRA. The purpose is to leverage the potential of knowledge contained in data which are presently scattered in different studies by merging them.

Interoperability can be broadly defined as “the ability of two or more systems or components to exchange information and to use the information that has been exchanged”^1^. In order to make efficient use of data, it is recommended to follow the Findable, Accessible, Interoperable, Reusable (FAIR) principles. These principles facilitate knowledge discovery of scientific data and their associated algorithms and workflows by humans and machines^2^. The use of interoperability standards that harmonize content and format of data, enhance the FAIRness of data, hence increasing their value. Semantic interoperability refers to the use of a common language to define concepts. This can be achieved by employing international terminologies and classifications that unambiguously define the meaning of concepts^3^.

The aim of our effort within ORCHESTRA was therefore to distinctly define each and every medical term, laboratory value, and other measurements and concepts used so that they can be uniquely identified and used by the partners to answer research questions^4^. Based on our experience with the standardization of the COVID-19–related German Consensus Dataset (GECCO)^5^ and similar to other FAIRification initiatives^6,7^, we pursued the semantic representation of the concepts by mapping them to standard terminology codes provided by organizations such as SNOMED International^8,9^, Logical Observation Identifiers Names and Codes (LOINC)^10,11^, Anatomical Therapeutic Chemical (ATC)^12^ and International Statistical Classification of Diseases and Related Health Problems (ICD)^13–15^.

Our endeavor to map over 2500 COVID-19-related concepts to standard codes enabled us to:

- Create a pool of standardized variables that can easily be merged with the same elements of new ORCHESTRA studies or with elements of external studies using the same terminologies and thus enhance their individual value.
- Identify common elements (core data set)^16^ between two ORCHESTRA studies which also included most elements of the third study; these common elements can now easily be merged without the need for further transformation.

The process of harmonization and standardization of data is demanding, but very crucial^17^ to share data especially within a large scientific community. It enables an efficient processing of information coming from many different sources. If health data are structured according to international standards, data are much easier to merge and analyze. Also, the efforts needed for data cleaning and pre-processing are reduced. An extensive employment of these standard terminologies across different projects would generally expedite data analysis while also providing research initiatives with a larger base of data.

## Results

### Harmonized data

The harmonization and standardization efforts led to the creation of two data dictionaries, one each for the Long-Term Sequelae (LTS) and Fragile Population (FP) study. The specific data elements defined for the Genomics study were included in the LTS and FP studies.

A dedicated working group managed the identification and linkage of patients and their related samples throughout ORCHESTRA.

**Fig. 1:**
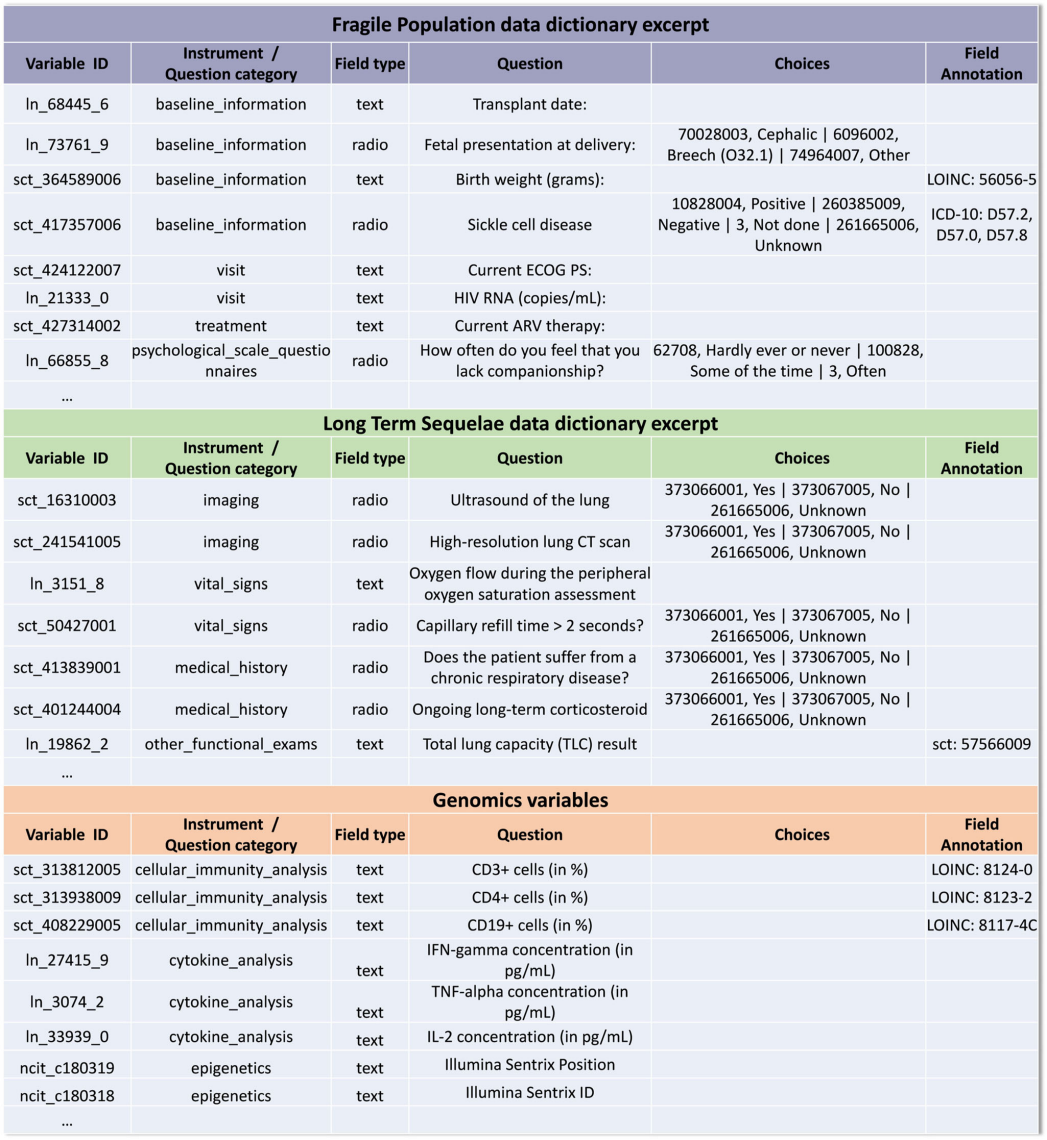
Excerpts of both the Long-Term Sequelae and Fragile population data dictionaries and selected Genomics variables. Examples of data dictionary elements with standard terminology codes incorporated in the variable IDs and answer (choice) IDs are shown as well as additional semantic representations of a concept that were added in the Field Annotation column. ECOG PS Eastern Cooperative Oncology Group Performance Scale, HIV RNA Human immunodeficiency virus ribonucleic acid, ARV Antiretroviral, CT Computer tomography, CD3 + cells Cluster of differentiation 3 positive T-cells, CD4 + cells Cluster of differentiation 4 positive T-cells, CD19 + cells Cluster of differentiation 19 positive B-lymphocytes, IFN-gamma Interferon gamma, TNF-alpha Tumor necrosis factor alpha, IL-2 Interleukin 2, pg/mL Pictograms per milliliter, ID Identifier.

Figure 1 shows excerpts from the two data dictionaries of the above-mentioned studies.

Table 1 shows the data elements that were standardized for the LTS study broken down into categories in form of REDCap® instruments. The LTS study’s eCRF comprises of 1118 data elements representing questions, descriptive text, and calculations divided into 30 different instruments. The greatest number of elements are included in the ‘Treatment’, ‘Biochemistry’ and ‘Symptoms’ categories with 179, 126, and 97 elements respectively.

**Table 1:** Variables defined and used in the LTS study.

Variable category/REDCap® instrument	Variable count
Treatment	179
Biochemistry	126
Symptoms	97
Questionnaires	73
Cytokine Analysis (EDTA^1^ Plasma)*	70
New Medical Events	61
Serology Tests (Serum Plasma)*	60
SARS-CoV-2 Vaccination	39
Imaging	36
Socioeconomic Questionnaire	36
Viral Variants And Respiratory Microbiome (Np Swab^2^)*	34
Vital Signs	31
Physical Examination	29
Epigenetics Methylation (EDTA Whole Blood)*	28
Other Functional Exams	28
Medical History	26
Human Genomics (EDTA Whole Blood)*	25
Cellular Immunity Analysis 1 (PBMCs^3^ 1)*	19
Cellular Immunity Analysis 2 (PBMCs 2)*	19
Fragile Population	17
Admission	13
6-Minute Walking Test	11
COVID-19 Complications	9
Demographics	9
Inclusion Criteria	9
Microbiological Tests	8
Outcome	8
IFNgamma Analysis Serum Plasma*	7
COVID-19 Severity	6
Intestinal Microbiome Stool or Rectal Swab*	5
**Total number of variables**	**1118**

Variables are broken down by informational category corresponding to the REDCap® instrument. The asterisk (*) denotes REDCap instruments that were defined by the Genomics study. ^1^Ethylenediaminetetraacetic acid. ^2^Nasopharyngeal swab. ^3^Peripheral blood mononuclear cell.

Table 2 shows the data elements contained in the data dictionary of the FP study by category. A total of 1440 data elements consisting of questions, calculations and descriptive fields were defined by the study team and harmonized thereafter. The greatest number of questions was specified for the ‘Adverse Events Related to Anti-COVID-19 Vaccine’ instrument with a total of 210 elements, followed by 188 elements for ‘Baseline Information’ and 179 elements in the ‘COVID-19 Treatment’ instruments. Subject matter experts have created a ‘socioeconomic questionnaire’ for use in the LTS and FP studies. The adult version of the questionnaire comprises of 36 elements whereas the child version is more compact and contains only 15.

**Table 2:** Overview of the variables defined and used in the Fragile Population study.

Variable category/REDCap® instrument	Variable count
Adverse Events Related To Anti-COVID-19 Vaccine	210
Baseline Information	188
COVID-19 Treatment	179
Biochemistry	134
Visit	98
COVID-19 Symptoms	97
Treatment	86
Cytokine Analysis (EDTA Plasma)*	68
New Medical Events	61
Socioeconomic Questionnaire	51
*(Adults: 36 variables|Children: 15 variables)*	
Comorbidities	42
Epigenetics Methylation (EDTA Whole Blood)*	31
Psychological Scale Questionnaires	31
Serology Tests (Serum Plasma)*	28
SARS-CoV-2 Vaccination	21
Biometric Parameters	17
Cellular Immunity Analysis 1 (PBMCs 1)*	15
Vaccination	15
COVID-19 Admission	13
Outcome	10
Viral Variants And Respiratory Microbiome (Np Swab)*	10
COVID-19 Complications	9
Microbiological Tests	8
COVID-19 Severity	6
Demographics	6
Inclusion Criteria	5
Imaging	1
**Total number of variables**	**1440**

Variables are broken down by informational category corresponding to the REDCap® instrument. The asterisk (*) denotes REDCap instruments that were defined by the Genomics study.

### Core data set

The assignment of unique names using standard terminology codes to the variables in both the LTS and the FP studies (including genomics data) enabled us to identify 743 common data elements. Since the standard codes are used as variable names, all 743 common elements between the two studies have the same variable names.

Similar to the data dictionary, the core data set consists of a spreadsheet listing the core data elements (CDEs) and their metadata. Additionally, besides the informational category of each data item, it records the clinical study that it was defined for and whether or not the element belongs to a predefined questionnaire. This information is very useful for example when submitting entire questionnaires to SDOs. The core data set, just like the data dictionaries, are constantly updated with new codes once they are available.

**Fig. 2:**
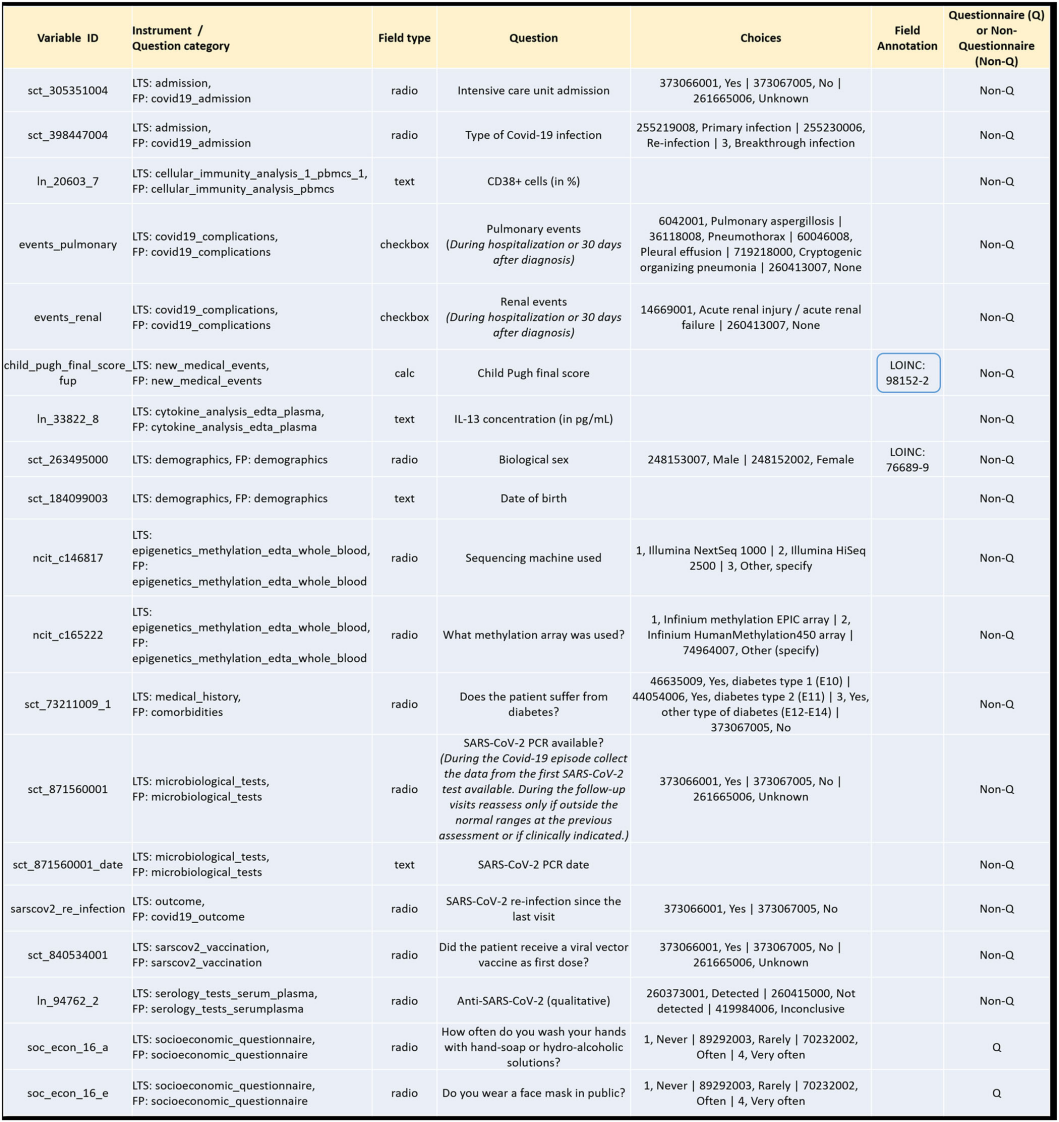
Excerpt of the Core Data Set. Examples of common elements from the LTS and FP studies. CD38 + cells Cluster of differentiation 38 positive immune cells, IL-13 Interleukin 13, PCR Polymerase chain reaction.

The blue box in Fig. 2 highlights an instance of a variable for which no international terminology standard code existed when the mapping started, but for which a LOINC code was released later, following a term request to the SDO. The new LOINC code was subsequently added to the Field Annotation.

Table 3 shows how many common elements per informational category were identified between the LTS and FP studies that have been analyzed.

**Table 3:** Overview of common variables used as part of the metadata for the Long-Term Sequelae study’s and the Fragile Population study’s electronic case report form respectively.

Variable category/REDCap® instrument	Variable count
Treatment	179
Biochemistry	126
Symptoms	97
Cytokine Analysis (EDTA Plasma)*	68
New Medical Events	61
Socioeconomic Questionnaire	36
Epigenetics Methylation (EDTA Whole Blood)*	31
Serology Tests (Serum Plasma)*	28
Vital Signs	19
SARS-CoV-2 Vaccination	16
Admission	13
Cellular Immunity Analysis 1 (PBMCs 1)*	11
COVID-19 Complications	9
Fragile Population	8
Outcome	8
Viral Variants And Respiratory Microbiome (Np Swab)*	8
COVID-19 Severity	6
Microbiological Tests	6
Medical History	5
Inclusion Criteria	4
Demographics	3
Imaging	1
**Total number of variables**	**743**

Variables are broken down by informational category corresponding to the REDCap® instruments. The asterisk (*) denotes REDCap instruments that were defined by the Genomics study.

The CDEs that make up the ORCHESTRA core data set comprised of 707 variables that contain stand-alone questions and their respective value sets (answers) and 36 variables that belong to questionnaires and their value sets (Table 4). Some questions are closed-ended and contain a fixed list of permissible answers, others are open-ended and allow open answers in form of text strings or numbers. Within the stand-alone questions, 441 variables are open-ended, another 5 variables replace value sets with formulas used to calculate scores and 6 variables contain descriptive text that does not have a corresponding value set. Descriptive text CDEs are an artifact specific to REDCap® and are means to provide instructions for the person entering the patient data. In the subset of variables that are part of questionnaires, 8 variables are open-ended, and 27 variables have value sets comprising fixed parameters.

**Table 4:** Overview of all common variables identified as core data elements for the ORCHESTRA core data set.

Variable value set types	Count of variables
Non-questionnaire questions:	707
with fixed value sets	255
with free text	441
with calculations	5
descriptive text	6
Questionnaire questions:	36
with fixed value sets	27
with free text	8
with calculations	0
descriptive text	1
**Total**	**743**

Variables are listed based on their value set types and whether they are part of a questionnaire.

**Fig. 3:**
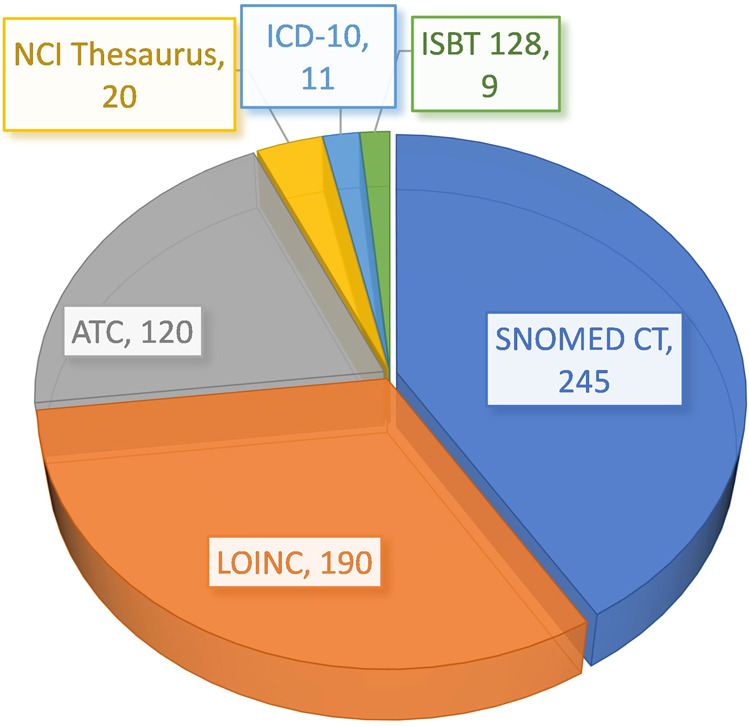
Unique standard codes. Overview of unique codes from recognized international standard terminologies and classifications assigned to common variables used in the LTS and FP studies’ electronic Case Report Forms.

Figure 3 details which international codes were assigned to represent the CDEs. The total number of codes is lower than the total number of CDEs because for some of the eCRF questions no appropriate codes could be assigned, either because the concepts contained in the variable were too complex or because no standard codes were available as of yet.

### Submissions

Submission to the most pertinent SDOs was evaluated for the data elements for which no corresponding international code could be found. Out of a total of 2558 variables and related answer lists, 125 concepts (57 stand-alone concepts and 68 items of Assessment scales/Questionnaires) were submitted to the SDOs SNOMED CT, LOINC, and NCI.

Table 5 shows the new concepts that were submitted to SNOMED CT and for which feedbacks are awaited.

**Table 5:** Healthcare concepts used in ORCHESTRA studies that were submitted to SNOMED CT for new code assignment.

Concept (Fully specified name)	Submission status	New code status
Ultrasonography of Lung abnormal	submitted to SNOMED CT	waiting for reply
Lung B-lines	submitted to SNOMED CT	waiting for reply
Air bronchogram	submitted to SNOMED CT	waiting for reply
Ground glass opacity	submitted to SNOMED CT	waiting for reply
Filtering face piece mask 2	submitted to SNOMED CT	waiting for reply
Filtering face piece mask 3	submitted to SNOMED CT	waiting for reply
SARS-CoV-2 breakthrough infection	submitted to SNOMED CT	waiting for reply
Lockdown	submitted to SNOMED CT	waiting for reply

The process of submitting concepts to LOINC was started for all eleven questionnaires used within the LTS and FP studies and for 30 serology concepts defined in the Genomics study (Table 6). Out of these, submission has been completed and new codes were created for six questionnaires whereas for one we are still waiting for codes. There is an ongoing effort to obtain authors’ permissions to finish the submission of the four remaining questionnaires. In addition, LOINC codes were also received for the submitted serology variables.

**Table 6:** Questionnaires and serology concepts used in ORCHESTRA studies that were submitted to LOINC for new code creation.

Questionnaire	Submission status	New code status
Post-COVID-19 Functional Status (PCFS) Scale^18^	submitted to LOINC	new codes received ✓
mMRC (modified Medical Research Council) Dyspnea^19,20^	submitted to LOINC	new codes received ✓
Socio-economic Questionnaire (Adults)	submitted to LOINC	new codes received ✓
Socio-economic Questionnaire (Children)	submitted to LOINC	new codes received ✓
Child-Pugh Score^21^	submitted to LOINC	new codes received ✓
COVID-19 clinical status by WHO classification^22^	submitted to LOINC	new codes received ✓
36-Item Short Form Health Survey questionnaire (SF-36)^23^	submitted to LOINC	investigating copyrights
Impact of Event Scale – Revised^24^	launched submission to LOINC	investigating copyrights
Maslach Burnout Inventory (MBI) questionnaire^25^	launched submission to LOINC	investigating copyrights
Abbreviated Profile of Mood States (POMS)^26^	submitted to LOINC	waiting for codes
Five Facet Mindfulness Questionnaire (FFMQ)^27^	submitted to LOINC	investigating copyrights
Non-questionnaire concepts	Submission status	New code status
Cytokines (30 values)	submitted to LOINC	new codes received ✓

Terms provided by the NCI Thesaurus were used to code data elements that contained the highly specific genetic information defined by the Genomic study and included in FP and LTS studies as shown in Tables 1 and 2. Table 7 shows the concepts that were submitted and received coding by NCI.

**Table 7:** Details of the 15 concepts that were submitted to NCI to request creation of codes and the newly created codes.

Concept	New NCI Code
Insert Size	C180312
Read Length	C153362
Read Group Identifier	C180313
Platform Unit	C180315
Nucleotide Sequence Sample Name	C180316
Nucleotide Sequencing Kit	C180317
Quality Control	C15311
Illumina Sentrix ID	C180318
Illumina Sentrix Position	C180319
Nucleotide Sequencing Plate ID Number	C180320
Pangolin Lineage Nomenclature	C180321
Pangolin Database Version Identifier	C180322
Nextstrain Clade	C180323
GISAID Accession ID	C180324
Sequencing Platform Name	C172274

In total, 92 new international standard codes (comprising 42 single concepts and 50 questionnaires/assessment scales items) relevant to COVID-19 research and beyond have been created as a result of our efforts.

**Fig. 4:**
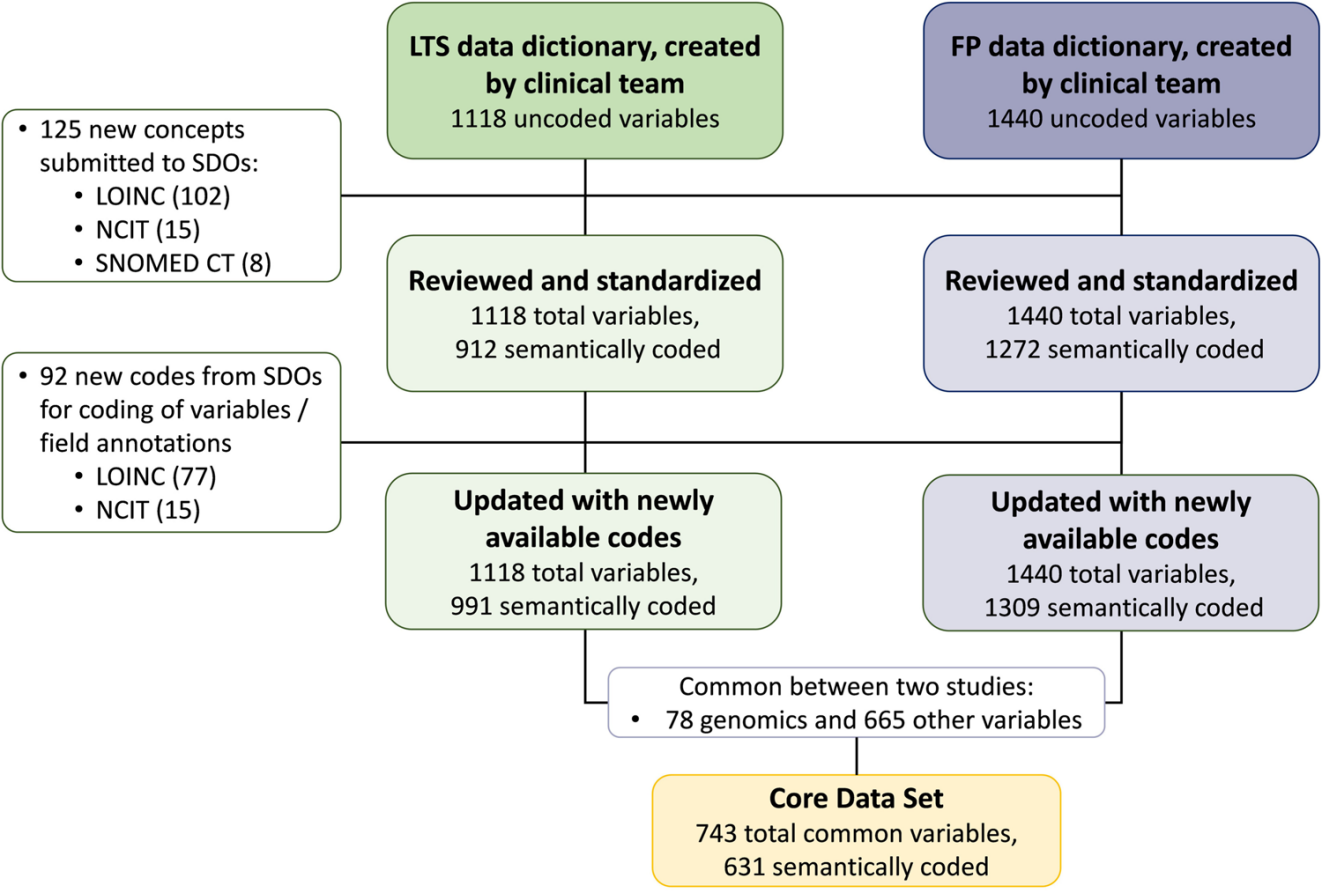
Overview of harmonized data and submissions to standard developing organizations. The diagram shows a summary of the variables used and semantically coded in the case report forms of the LTS and FP clinical studies and the concepts submitted for coding to standard developing organizations.

Figure 4 summarizes the results described in the paragraphs above.

## Discussion

The effects of the COVID-19 pandemic have highlighted the need to gather new scientific insights into the pathology of the disease including its progression, effects on different population groups, vaccination monitoring, and long-term impact. Several SARS-CoV-2-related studies^28–30^ collected data and added to the growing body of knowledge about the disease^31–33^. ORCHESTRA has a multidisciplinary approach to estimate the clinical and social aspects of the burden of COVID-19 across different health care systems while improving comparability of data. The project includes 26 partners from 14 different countries in Europe in addition to 3 partners from non-European regions such as India, South America, and Africa. However, data collected often vary in structure and format, moreover are stored in different databases that are not interoperable, thus reducing the potential to answer important research questions.

Starting with three ORCHESTRA studies, we have addressed this problem by employing healthcare-specific interoperability standards that unambiguously identify variables and make health data more transferable to and interpretable by different IT systems and applications^34^. As a result, we obtained all possible variables mapped to their respective domain-relevant standard codes which were also incorporated into variable IDs. This pool of standardized variables can now be used to facilitate merging of data of the examined studies with:

- New ORCHESTRA studies that include any of those elements
- Ongoing ORCHESTRA studies that, after being mapped to standard terminologies, are found to have matching variables
- Any study that makes use of the same variables identified by the same standard terminologies

Additionally, introducing a standardized ID allowed us to identify 743 common elements between two of the three studies under consideration. Common data elements mainly fall into the ‘Treatment’, ‘Biochemistry’ and ‘Symptoms’ categories. In addition, most elements of the Genomics study were included in the pool of common data elements as well. In fact, the Genomics study is much smaller in terms of number of variables than the other two. But by focusing on sample-related genomics information, it enables a much-pursued deeper investigation of the SARS-CoV-2 virus and its variants.

The result is consistent with the notion that both studies would explore similar treatments that patients have received and would also request information about the same types of laboratory values and blood panels. Partners were very committed and motivated to increase possibilities of data sharing and analysis across studies and therefore made a real effort to adapt data definitions in each study to converge as much as possible on a common data set. Emphasis was also put on harmonizing the collection of data concerning symptoms experienced by patients.

Data collected by the LTS and FP studies for these 743 common data elements could be immediately merged and used for analysis without requiring any further transformation. The first analysis results for ORCHESTRA are expected in the first half of 2022. The harmonization work done so far will be utilized for other ongoing ORCHESTRA studies: each study’s data elements will be progressively mapped to standard terminologies, and new common elements will be identified and included in the COVID-19 core data set (CDS).

The use of CDEs to harmonize data originating from different studies has already been described^35,36^ as a method to merge data in different medical disciplines. In their work, Meeuws et al.^37^ show how the presence of CDEs on Traumatic Brain Injury has enabled a high level of harmonization in data from three different large multi-centric studies. The CDEs described usually relate to those provided by the American National Institute of Neurological Disorders and Stroke (NINDS) for a range of diseases (https://www.commondataelements.ninds.nih.gov/), but their mapping to standard terminologies is not always available. In our approach we are building the COVID-19 CDS along with the harmonization and mapping process. By doing so, we will on the one hand expand the pool of COVID-19-related standardized variables, and on the other hand we will continue to identify common data elements between studies and update our CDS.

In a similar approach, the development of a data model that includes mapping to SNOMED CT, LOINC, ICD-10, has been described for the Utah Newborn Screening (NBS) Program to improve the data exchange between healthcare providers and NBS programs. This leads to a reduced dependency on proprietary laboratory information systems^38^.

A major challenge when harmonizing epidemiologic data is to fully understand the definition of the variables that need to be combined. The meaning of variable names and descriptions is not always understood in the same way from one person to another. The use of specific terminology standards sometimes implies a higher level of detail in the definition of the concept. For this reason, changes might be required in some data definitions to include more details. For example the variable “Hemoglobin” is represented in LOINC by different codes depending if the value is expressed in “mmol/L” or in “g/dL”; it is therefore necessary to specify the unit in the variable definition. This further specification of the information to be collected avoids different interpretations of the concept, implicitly supports the homogenization of the information, and ultimately improves the quality of the data. However, it is difficult to make changes to variables when data collection has already started. If data harmonization is not performed upfront, it needs to be done after data collection with a much bigger effort. As Rolland et al. observe “even with substantial documentation, it can be challenging to understand data that someone else has collected, without engaging in time-consuming conversations with the original data collectors”^39^. Additionally, the quality of data, integrity, completeness as well as its ability to be traced can be compromised in this process.

Generally, to achieve a wider implementation of interoperability standards, political barriers in sharing data must be removed, goals should be aligned, and extensive collaboration between research and healthcare organizations is needed^40^. The process to receive permission for reuse of data should also be well defined and transparent^41^. The pandemic could be an opportunity to broaden the use of CDEs beyond specific implementations by engaging the broader research and healthcare communities. Strong public advocacy is needed for the broad use of standards in healthcare research and in all data collections; this would speed up the extraction of knowledge from data by facilitating the exchange and merge of the information. In ORCHESTRA, an important result was achieved in identifying a first COVID-19 CDS that could be useful also for other external COVID-19 studies. However, the harmonization work within ORCHESTRA continues as other data collection protocols are being developed.

While it is important to make use of international healthcare standards, it is also desirable to actively contribute to the standardization efforts by submitting new concepts for coding when they are not already included in the terminologies. The acceptance by SDOs of the submitted concepts that were needed for use in the ORCHESTRA studies is a great achievement that resonates outside of this particular use case since it constitutes an active contribution to standardization. Since the COVID-19 pandemic, there have been new terms emerging every day. From specific laboratory test names to terms related to social distancing, there are many more new expressions, which were introduced during this period. In order to facilitate research on emerging infectious diseases, it is of particular importance to enrich the international standard terminologies with the new terms, by actively submitting them to the SDOs. Such new concepts and codes become thus available internationally and can be used globally to identify uniquely the same concepts also in other projects. Moreover, the two socio-economic questionnaires that were developed by experts within ORCHESTRA and have been submitted to LOINC could become reusable and available worldwide for other COVID-19-related studies and thus potentially provide comparable data on the socioeconomic effects of the infection. On the other hand, when new more specific codes become available, it is likely that some other codes already in use become obsolete or too generic. For this reason, it is important to reference also the new codes in the metadata.

A thorough evaluation of the use of LOINC in pathology laboratories showed some of its limitations and challenges^42^. Among other results, mapping inconsistencies especially in the properties of methods used were discovered between laboratories. The lack of explicit hierarchy in LOINC prevents the easy identification of related terms thus making the mapping process for granular differences in tests more challenging. We tried to mitigate the problem by working together with the experts and asking to confirm the methods used in dubious cases.

During the mapping process, we realized that molecular genetic diagnostics are not yet properly represented in the international terminologies considered, probably due to the new and fast-evolving methods and discoveries in this field. The knowledge of genes and genomes is indeed one of the rapidly growing areas of biomedical research. The high number of genetic tests with diverse attributes, involving over 20.000 genes, is posing new challenges to keeping the terminology systems up to date^43^.

However, there is a continuous process allowing for improvement proposals to LOINC which are then implemented, such as the identification of LOINC’s implicit hierarchies^44^. Additionally, an implementation guide for structured reporting of genetic tests was published by the HL7 Clinical Genomics Working Group containing guidance on how LOINC codes are to be used, and details on variable linkage to specific lists of permissible answers^45^.

For information related to genetics, the National Cancer Institute Thesaurus turned out helpful in covering concepts that were not included in LOINC nor in SNOMED CT, probably due to the primary role of genomics in current cancer research.

The work described here shows that the combined use of standard terminologies is the best solution for embracing the different categories of information collected.

In summary, our work aimed at enhancing semantic interoperability within the international research community in the field of COVID-19 by making use of international standard terminologies and classifications. Data collected in different studies using the same CDEs can be merged directly without need for further transformation, thus accelerating research results.

Our pool of standardized variables can also be used beyond the project’s borders by other research initiatives.

Many aspects of the SARS-CoV-2 infection were still widely unknown at the beginning of our work and a language to describe them had not been fully built. Thus, we contributed to COVID-19 research by submitting new concepts (over 100 concepts in total) for coding to SDOs so that they could become available for research worldwide. Our approach expedites research collaborations and processing of results.

## Methods

The work presented here does not involve human participants or data. It is based on the analysis of dataset definitions from ongoing or new studies coordinated by the ORCHESTRA partners, for which individual ethical approvals were obtained. In this study we are only investigating metadata, and no Human Subjects Research is involved.

### Harmonization of studies

The process of harmonization started with three clinical studies in ORCHESTRA. The first study focuses on investigating long-term Sequelae after COVID-19 infection, hereafter referred to as the “Long-Term Sequelae” (LTS) study. The second prospective study focuses on patients considered fragile, hereafter referred to as the “Fragile Population” (FP) study. FP is an already ongoing study on post-vaccination monitoring and is also participating in the LTS study with fragile patients with history of prior COVID-19 diagnosis. For the FP study, we worked on harmonization of retrospective and prospective data concurrently. The third ORCHESTRA study, hereafter referred to as the “Genomics” study focuses on biobanking of patient samples, genomics, and viral–host interaction analysis.

**Fig. 5:**
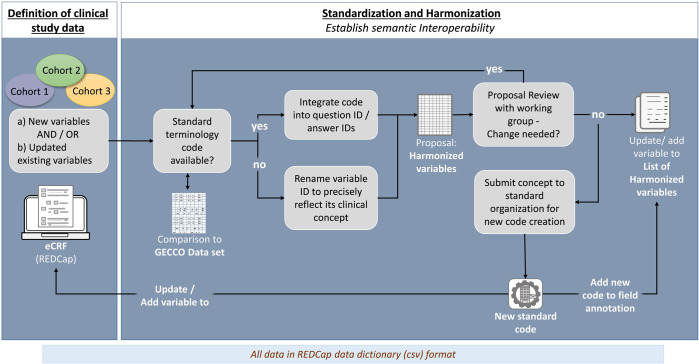
Standardization and harmonization workflow. The diagram shows the different steps of the standardization and harmonization process in ORCHESTRA.

Figure 5 depicts the workflow followed to standardize and harmonize the three prospective studies in ORCHESTRA. The clinical study teams for all three studies were the starting point for the activities. They defined the clinical concepts of their study protocols within a data dictionary that is used in the REDCap® electronic data capture (EDC) system to record patient data; subsequently, they submitted their data dictionaries to our standardization team.

In the first instance all variables received from the LTS study were new and not standardized. Subsequently, we received updated variables in several iterations. This included changes to previously defined elements, removal of data elements, and addition of new data elements that had to be considered for the standardization and harmonization process. Our first activity was to review the received data dictionary and to assess whether the variables could be mapped to international standard terminology codes.

As part of that activity, the study data elements defined by clinical partners were compared with GECCO to identify information that had already been standardized i.e. associated with international standard terminologies and classifications. More information can be found in the “GECCO Data Set” section of the Supplementary Material.

**Fig. 6:**
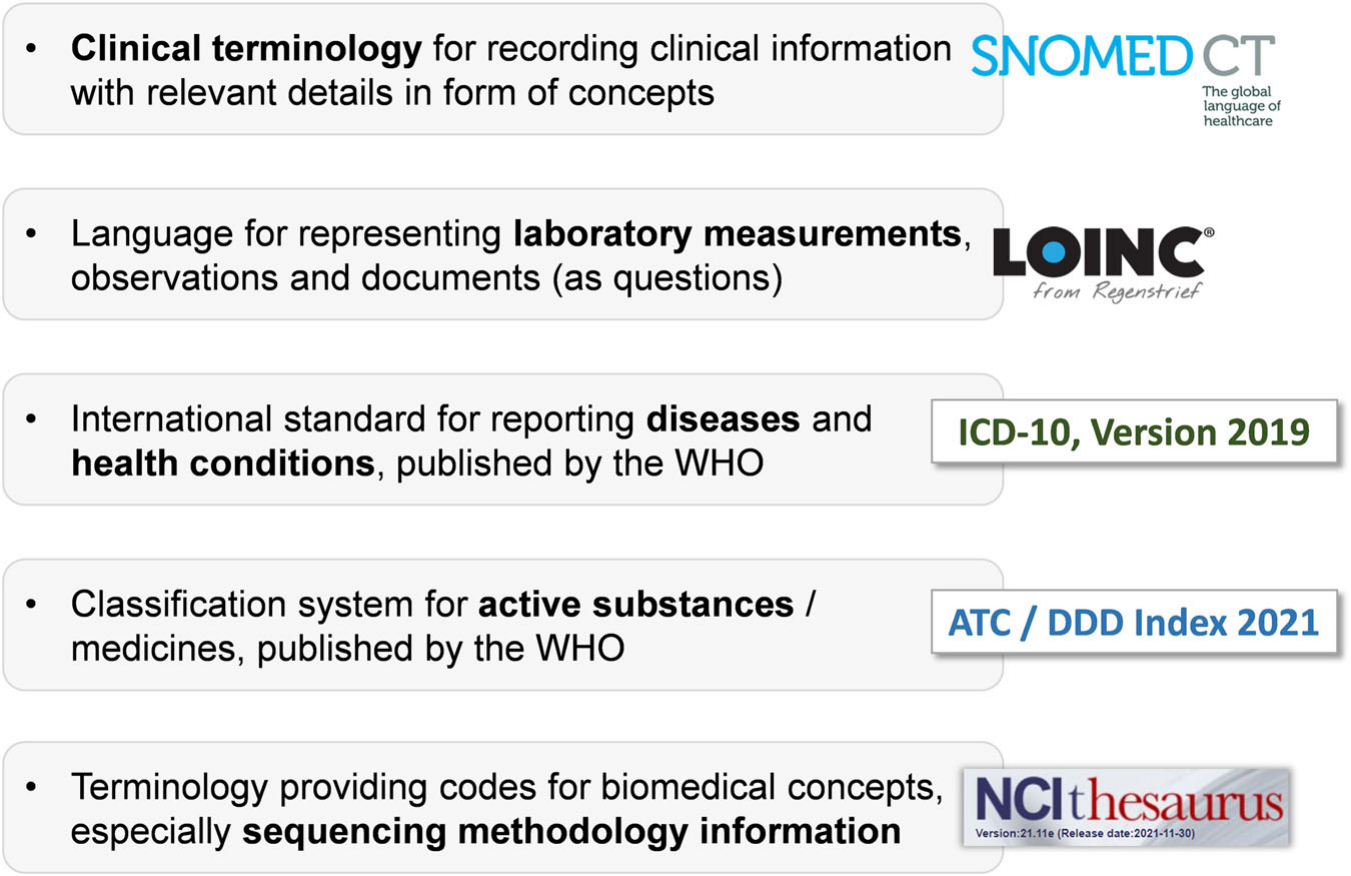
Standard Terminologies. Overview of the main terminologies used to code ORCHESTRA variables to ensure semantic interoperability.

If the variable was not already included in GECCO and therefore needed to be newly standardized, we chose the most appropriate standard terminology (Fig. 6) to be used for its representation enabling semantic interoperability.

For example, for general clinical concepts we used SNOMED CT because it is the world’s most comprehensive clinical healthcare terminology^46^. Codes were searched using the SNOMED CT Browser’s International Edition^47^ and assigned to data elements wherever appropriate. Laboratory values, vital signs, and questionnaires were mapped to LOINC codes that were selected using the LOINC search browser^48^. LOINC played a very important role in defining the SARS-CoV-2 specific laboratory tests referenced in the ORCHESTRA studies’ electronic Case Report Forms (eCRFs);^49,50^ it was chosen because it is a widely used terminology standard for health measurements, observations, and clinical documents^51^. For variables that aimed at collecting information on medication use, ATC codes were selected using the WHO’s ATC/DDD Index 2021^52^. Data that provided genetic, epigenetic, and sequencing information were generally assigned codes chosen through the use of the NCI Term Browser^53^. The WHO’s search browser provided means of finding appropriate ICD-10 codes to assign to data elements detailing diseases and disorders. To code convalescent plasma treatment, the ISBT 128^54,55^ standard’s lookup tools were used to assign codes to represent the respective data elements. Further information on international standard terminologies can be found in the Supplementary Material.

We firstly analyzed all 851 variables used in the LTS study and mapped them to the appropriate standard concepts whenever possible. The process was performed for both components of each variable: the question and its respective answers /value sets (if value sets were defined).

Whenever an appropriate code was available in a standard terminology to represent the clinical concept contained in a question or in the answer(s), we integrated it directly into the variable ID or answer ID respectively. The coding of variables along with any outstanding issues and questions were discussed in review meetings with the working groups. When a standard code for a clinical concept within the data dictionary did not exist, we started a submission process at the SDOs to request the creation of new specific codes for the concepts.

Due to the fact that submissions for new codes can take up to several months, we could not always integrate the codes in the variable names right away. In this case, we renamed the variable ID to a name that could represent the concept as closely as possible so that it could be promptly returned to the clinical teams and uploaded into REDCap® for immediate use. When a requested code was created and made available by the SDOs, it was integrated as field annotation in the REDCap® data dictionary.

A similar process was performed for the FP study for which we analyzed over 1250 variables and their respective answers and for the 267 sample-related variables of the Genomics study.

### Coding within data capture tool

A dedicated instance of the EDC system REDCap® was provided by the Italian University Consortium CINECA (https://redcap-dev.orchestra.cineca.it) to support the definition and collection of the variables as well as the process of standardization and harmonization through the use of the data dictionary. The data dictionary is a specifically formatted spreadsheet with a.csv extension containing the metadata used to construct electronic data collection instruments through its upload in REDCap®. It is divided in several columns, each containing a different type of metadata. Two data dictionaries, for the three considered studies, were built to hold all the clinical study variables, namely questions and value sets to be collected through patient interviews at study visits. Clinical subject matter experts assigned different categories to the data by grouping them into ‘instruments’ according to the informational domains. These categories, defined as well in the data dictionaries, ranged from variables pertaining to patient admission, demographics, functional and physical exam results, clinical outcomes, symptoms, vaccination, imaging, samples, and socioeconomic situation. REDCap® accounts were provided both to the partners involved in the standardization process and to the scientific team defining the data elements to collect. This made it possible to interactively update the data dictionary while working on setting up the eCRFs for data collection. Supplementary Fig. 1 shows an example of the dataset Codebook, the human-readable version of the data dictionary. More information on REDCap® can be found in the dedicated section of the Supplementary Material.

Standardization was performed on the variables by identifying the corresponding standard codes and entering them in the data dictionary. In particular, we inserted them in the mandatory field for the variable name preceded by a prefix to identify the terminology used (e.g. sct for SNOMED CT or ln for LOINC) for every data element created in the eCRF for which a standard code was available. Analogous to the variable ID that identifies an element, every answer choice is identified with an ID code as well.

Where appropriate, concepts contained in the answers were also standardized and the respective international standard code was integrated into the answer ID.

**Fig. 7:**
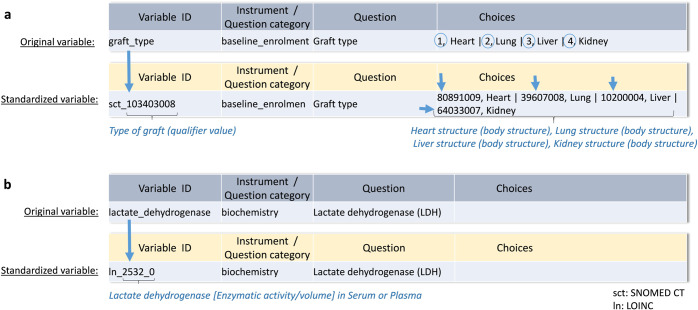
Assignment of standard terminology codes to variable and answer IDs. **a** Assignment of SNOMED CT codes to represent the clinical concept of the question in the variable ID and the concepts contained in the answers as codes in the answer IDs. **b** Assignment of appropriate LOINC code representing the laboratory value lactate dehydrogenase to the variable ID of the respective question in the data dictionary.

Examples of this procedure are shown in Fig. 7. where the concepts “Graft Type” and “Lactate dehydrogenase” were respectively mapped to the SNOMED CT code *103403008*|*Type of graft (qualifier value)|* and to the LOINC code *2532-0 Lactate dehydrogenase [Enzymatic activity/volume] in Serum or Plasma* and then integrated into the Variable IDs. In Fig. 7a, it is possible to see how standard codes were also associated to the answer list.

REDCap® also provides the option to add an annotation to each element by use of the Field Annotation column that is part of the data dictionary. The Field Annotation was used to list other possible standard codes when available.

**Fig. 8:**
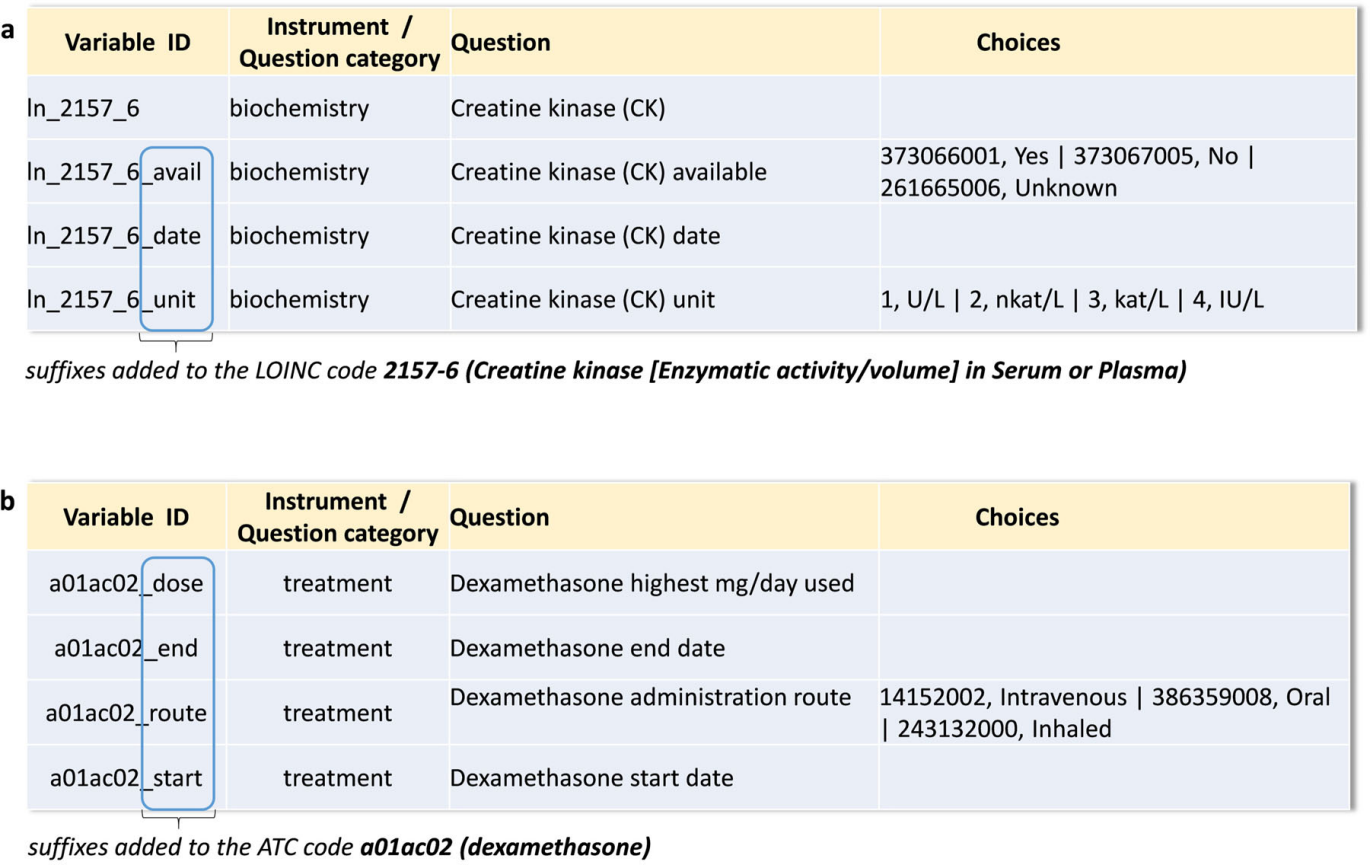
Incorporation of suffixes into the standardized variable names of data used in the Long-Term Sequelae and Fragile Population studies. **a** Overview of suffixes used as part of the variable names for the laboratory component creatine kinase which was coded with the appropriate LOINC code. **b** Overview of suffixes added to the ATC code for dexamethasone as part of the variable names of the related data elements. U/L: Unit per liter, nkat/L: Nanokatal per liter, kat/L: Katal per liter, IU/L: International unit per liter.

For many of the laboratory values, information related to a specific test was collected across several variables, covering details such as whether a test result was available, the test result itself, the date of the test, and unit of measurement. In order to ensure the link between the five details about one clinical test would remain obvious, we re-used the international code assigned to the variable ID of the actual test result and added suffixes to the variable IDs representing the additional informational domains. For example, the variable ID “ln_2157_6” contains the LOINC (abbreviated with ‘ln’) code 2157-6 for ‘Creatine kinase [Enzymatic activity/volume] in Serum or Plasma’. The additional four connected variables, ‘ln_2157_6_avail’ for the availability of the test information, ‘ln_2157_6_date’ for the date of the test, ‘ln_2157_6_unit’ to clarify the unit of measurement, all contain suffixes in addition to the standard code to help maintain the connection to the central variable, the test result in itself (Fig. 8a).

When it came to variables that were mapped to ATC codes to describe a medication regimen, we decided to incorporate suffixes in the variable IDs to enable us to reflect clinical concepts like start and end date or dose and route of treatment. This was done by adding the suffixes “_start”, “_end”, “_dose” or “_route” to the variable ID (Fig. 8b).

### Common data elements

Harmonization of data collection across clinical studies was based on the identification of CDEs^56,57^.

CDEs can be defined as set “of a precisely defined question paired with a specified set of responses to the question that is common to multiple datasets or used across different studies”^58^. We followed the American National Institutes of Health’s (NIH) approach in classifying data elements^59^ and put an emphasis on only selecting data elements with high priority to define the core data set. Elements common to both the Long-Term Sequelae and Fragile Population studies were considered ‘High Priority’. Elements that were included in only one of the study data sets were not considered highly relevant and remained in the study-specific data sets. The core and the study-specific data sets were published on the public platform Art-Décor®^60^ which is a free tool that facilitates the modeling of data sets with their bindings to terminologies.

An important part of the harmonization was to clarify the meaning of variables, ensure an unambiguous wording, and promote the use of the same variables across both studies. Meticulous efforts were undertaken by the partners to try to format them exactly the same way in terms of content, phrasing, and answer value sets. In this way, the variables could be assigned the same codes for the different partners, thus also facilitating the merging of data for research purposes.

To achieve that goal, we suggested adjustments to clinical partners when we identified variables that contained the same clinical concept in the question across both studies but where each study had slightly different answers in the value set of the question. The final decision to follow our suggestion rested with the working group who weighted the option against their clinical objectives.

**Fig. 9:**
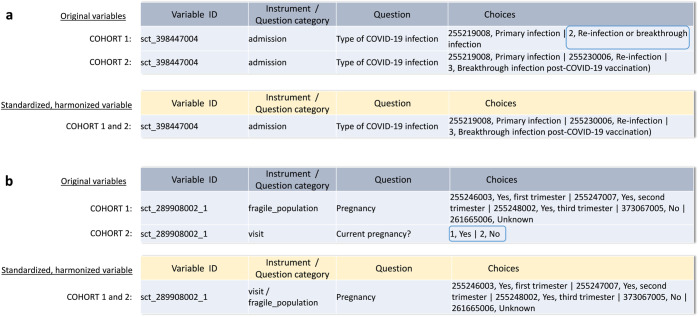
Harmonization of value sets for two common variables. **a**, **b** show how different answer value sets between two clinical studies in ORCHESTRA converged to maximize precision and interoperability.

One example of such a proposed change is the variable concerning the type of COVID-19 infection. Within the FP study, three answers were defined to the question: ‘primary infection‘, ‘re-infection ‘and ‘breakthrough-infection post-COVID-19 vaccination‘. In contrast, the LTS study only offered two answers as part of the variable value set: ‘primary infection‘ and ‘re-infection or breakthrough infection‘. As the three option value set was most precise, we suggested the LTS study adapted their variable to match the FP study’s. The proposal was accepted and the variable was added to the core data set for ORCHESTRA (Fig. 9a).

In another case, we proposed to partners from the Fragile Population study to adapt their pregnancy status variable’s value set to match the one defined in the LTS study. This would change the value set from yes or no answers to answers that also incorporate information about the current trimester if a pregnancy was confirmed present (Fig. 9b).

Some concepts were identical between the two studies, but had different data collection time points. In this case, when naming the variables, a suffix was added to the standard unique code to identify the time point.

Genomics data variables were uniquely defined by the Genomics study team and were standardized following the same principles as the other two studies.

### Software

The original data set definitions received from clinical partners were analyzed in LibreOffice Calc Version 7.1.1.2. The core data set was developed in Microsoft Excel 2016. All graphs shown in this publication were created in Microsoft PowerPoint 2016 while tables were built in Microsoft Excel 2016. The version of REDCap® used for the work described was 12.0.1. The standard terminologies used are: SNOMED CT International Edition July 2021, LOINC 2.71 ICD-10. ATC/DDD Index 2021, NCIt Version 21.11.e. To look up codes we used International SNOMED CT Browser Version 2021-7-31, SearchLOINC Version 2.20, ICD-10 online search application Version 2019, NCI Term Browser Version 2.19.

### Reporting summary

Further information on research design is available in the Nature Research Reporting Summary linked to this article.

## Supplementary information

Supplementary Material

Reporting Summary

## Data Availability

The metadata definitions for the three data sets are publicly available on the standard-enabling platform ART-DÉCOR® (https://art-decor.org/art-decor/decor-project--orch-). The data sets in ART DÉCOR® receive periodic updates to reflect possible changes in the CRFs, consequently, they might differ from the ones we refer to in the present work. The Data Dictionaries that were created are stored in a repository online (https://cloud.orchestra-cohort.eu/s/HeycD4xY7TACxLX. Access to the files can be granted upon reasonable request.
