# Supplementary material for: Harmonization and standardization of data for a pan-European cohort on SARS- CoV-2 pandemic
Source: NPJ Digit Med. 2022 Jun 14;5:75. doi: 10.1038/s41746-022-00620-x (PMC9198067; doi:10.1038/s41746-022-00620-x)
Supplement: Supplementary file 1 — Supplementary Material [file 41746_2022_620_MOESM1_ESM.pdf]

SUPPLEMENTARY MATERIAL

International standard terminologies

Semantic interoperability plays a central role when trying to merge data from different sources, each describing concepts in their own way. It is therefore important to identify the best-suited and worldwide recognized terminologies for the different categories of healthcare concepts.

The standard terminology maintained by SNOMED International, called SNOMED Clinical Terms (CT) is particularly well-suited as a general-purpose language for advancing semantic interoperability in medicine and healthcare due to its vast repertoire of available codes and hierarchical structuring of concepts and the definition of relationships between them. For example, the subtype relationship defines one concept as a subtype of another. This enables efficient classification of a clinical condition by including references to a SNOMED CT concept with all its hierarchical "children" and further descendant subtype concepts[1,2]. It can be complemented by more domain-specific terminologies. LOINC, for example, is typically used for laboratory observations and assessment tools. Each LOINC code represents the "question" that forms the basis of a test or measurement.

A LOINC term is made up of several components representing different information: the substance entity or specimen that is being measured, characteristics of the analyte, the time interval over which the observation was made, the type of value (nominal, ordinal or quantitative) and optionally the method used for analysis.

The Unified Code for Units of Measure (UCUM) [3] is used for measurement units.

ICD-10, the ICD in its 10th version is published by the World Health Organization (WHO) and used for reporting diseases and health conditions" [4] for both clinical and research efforts. ICD codes define classifications not only for diseases but also for injuries

and disorders and are ordered hierarchically. Although a revision of ICD is already available from the WHO platform, ICD-11 will officially come into effect on 1 January 2022 and it will take quite some time before it becomes broadly used.

The WHO's ATC classification best represents medications [5]. ATC is also being used by the surveillance system of the European Centre for Disease Prevention and Control (ECDC) for example to monitor antimicrobial consumption [6].

For specific genetics investigations, following the approach of the Global Alliance for Genomics and Health (GA4GH) [7,8], the National Cancer Institute's (NCI) thesaurus (NCIt) [9] was found to offer the most complete terminology resources.

The use of plasma products is one of the many treatments [10,11] that have been investigated in an effort to combat severe COVID-19 infections. To represent the concept of convalescent plasma therapy, the ISBT 128 standard for medical products of human origin was selected [12].

All of these terminologies together can ensure that health data have a clear structure and unambiguous semantics.

GECCO Data Set

The GECCO (German Corona Consensus Data Set) research dataset on COVID-19 [13–15], standardized by the Charité, has been selected as reference for creating a core data set in ORCHESTRA. GECCO is part of the German COVID-19 Research Network of University Medicine (https://www.netzwerk-universitaetsmedizin.de), which aims to bundle the resources of German university hospitals to improve diagnostics and treatment of COVID-19 patients.

GECCO was developed using international health IT standards and terminologies for interoperable data exchange. In the development process of GECCO, the international project ISARIC-WHO CRF [16,17] was taken into account. Additionally, also data elements and the

corresponding value sets from relevant German projects were considered such as the German Pa-COVID-19 study [18], which investigates the pathophysiology of COVID-19 in a prospective patient cohort. Also the LEOSS [19,20] case registry was taken into account, a clinical patient registry for patients infected with SARS-CoV-2 initiated by the ESCMID Emerging Infections Task Force (EITaF) and the German Center for Infection Research (DZIF) and the German Society for Infectiology (DGI). The GECCO dataset was originally developed for use by the German university hospitals that partner to share their data for common analysis within a centralized platform as part of the CODEX (COVID-19 Data Exchange Platform) project (https://www.netzwerk-universitaetsmedizin.de/projekte/codex). However, since international standards and terminologies were used, the GECCO dataset can be extended to use cases beyond its original intention and also be applied in international contexts [21]. The GECCO FHIR profiles are also based on international work such as for example the International Patient summary (IPS) [22]. This ensures that the GECCO dataset can be re-used also internationally and thus supports interoperability. For this reason, it was possible to consider it as starting point for ORCHESTRA.

ORCHESTRA studies

In order to maximize potential insights that could be gained, the project ORCHESTRA includes SARS-CoV-2 infected and non-infected individuals of all ages and pre-existing conditions, focusing on at-risk populations of vulnerable individuals and healthcare workers. Patients with history of COVID-19 will be followed in the ORCHESTRA studies for the assessment of clinical, radiological, and psychological consequences up to 18-month after diagnosis of COVID-19 [23]. The inclusion of the fragile population will offer an opportunity to explore the impact of COVID-19 on frail or at-risk populations, who are usually not represented enough among the general population cohort. The fragile population cohort also

has the advantage of being well structured and established with routine follow-up visits and sample collections, thus facilitating the assessment of the COVID-19 long-term consequences as well as the monitoring of immune response to COVID-19 vaccination..

Long-Term Sequelae study

COVID-19 can result in long-term sequelae in adults, young adults and children without underlying chronic medical conditions, regardless of the severity of the acute infection. The terms ‘Long-Term Sequelae’ or ‘Long-COVID’ are used to refer to individuals who experience new or prolonged symptoms for more than 28 days after initial COVID-19 diagnosis [[24]]. In a telephone survey conducted by the Centers for Disease Control and Prevention among a random sample of 292 adults (≥18 years) who had a positive outpatient test result for SARSCoV-2 by RT-PCR, 35% of 274 symptomatic respondents reported not having returned to their usual state of health two weeks or more after testing [[25]]. The burden of COVID-19 long-term sequelae and the exact underlying pathophysiology mechanisms remain unknown [[26]]. Results from the follow-up of large cohorts are particularly needed to fully understand the characteristics and risk factors for SARS-CoV-2 infection long-term consequences.

From the point of view of social science, this study will allow investigating two largely unexplored issues. Firstly, there is a need to explore the determinants of adherence to preventive strategies, including non-pharmacological measures and vaccines. There is a particular lack of knowledge concerning individuals who could develop breakthrough infections after SARS-CoV-2 infection or vaccination and possible associations with viral variants and biomarkers. Secondly, there is also lack of knowledge about the strain and demands that SARS-CoV-2 pandemic puts on healthcare system resources across different settings such as hospitals, general practitioners’ practices, long-term care facilities, or on the

resource availability (e.g. beds, oxygen supplies, personal protective equipment, etc.). Parameters such as the length of hospital stay, frequency of follow-up visits, for example, have been projected at the start of the pandemic but the actual figures have not been openly disclosed and explored extensively yet.

Fragile population study

The ORCHESTRA fragile cohort consists of ten existing and five new cohorts of fragile patients from 11 European and 5 non-European countries, with approximately 14300 subjects. These will include pregnant women/newborns, children, patients with HIV infection, solid organ transplant recipients (SOT), patients with immunologic disorders and patients with Parkinson's disease.

Since the beginning of COVID-19 pandemic, several at-risk population groups have been identified regarding the susceptibility to SARS-CoV-2 infection, the associated clinical spectrum and outcome. They include pregnant women, pediatric patients, and immunocompromised hosts including SOT, hematopoietic stem cell transplant (HSCT) recipients, and patients with cancer.

Among pregnant women and children, asymptomatic or mild diseases have been frequently reported, and their role in the transmission of infection in community and hospital settings still needs further investigation [27–29]. On the other hand, a high impact of COVID-19 on morbidity and mortality has been described in elderly and immunocompromised hosts [30,31]. Optimization of prevention strategies, screening practices and therapeutic management is therefore recommended when dealing with fragile patients [32,33]. Epidemiological data are strongly needed to design further intervention trials and health policies.

Genomics study

Several different sample types will be analyzed within the ORCHESTRA study for the purposes of identifying human and viral genetic markers indicative of disease severity as well as to study immune responses over time in response to infection and immunization. Specifically, samples will be collected from patients with COVID-19 (including breakthrough and reinfection) to study both short- and long-term effects of infection on host immunity, respiratory and intestinal microbiome dynamics, as well as host and viral genetic determinants underlying infection. Additionally, samples will be collected from vaccinated fragile populations as well as vaccinated healthcare workers to study effects of vaccination on host immunity and respiratory and intestinal microbiome dynamics.

Samples collected within the framework of the ORCHESTRA study will in many cases be subjected to more than one type of analysis.

REDCap®

Study data were collected and managed using REDCap electronic data capture tools hosted within ORCHESTRA [[34,35]].REDCap (Research Electronic Data Capture) is a secure, web-based software platform designed to support data capture for research studies, providing 1) an intuitive interface for validated data capture; 2) audit trails for tracking data manipulation and export procedures; 3) automated export procedures for seamless data downloads to common statistical packages; and 4) procedures for data integration and interoperability with external sources. The REDCap® project was developed to provide scientific research teams intuitive and reusable tools for collecting, storing and disseminating project-specific clinical and translational research data. The following key features were identified as critical components for supporting research projects: 1) collaborative access to

data across academic departments and institutions; 2) user authentication and role-based security; 3) intuitive electronic case report forms (CRFs); 4) real-time data validation, integrity checks and other mechanisms for ensuring data quality (e.g. double-data entry options); 5) data attribution and audit capabilities; 6) protocol document storage and sharing; 7) central data storage and backups; 8) data export functions for common statistical packages; and 9) data import functions to facilitate bulk import of data from other systems. Given the quantity and diversity of research projects within academic medical centers, we determined two additional critical features for the REDCap® project: 10) a software generation cycle sufficiently fast to accommodate multiple concurrent projects without the need for custom project-specific programming; and 11) a model capable of meeting disparate data collection needs of projects across a wide array of scientific disciplines.

REDCap® accomplishes key functions through use of a single study metadata table referenced by presentation-level operational modules. Based on this abstracted programming model, studies are developed in an efficient manner with little resource investment beyond the creation of a single data dictionary. In the Supplementary Figure 1, a section of the REDCap 'read-only' version of the much larger Data Dictionary for the project is shown.

The concept of metadata-driven application development is well established, so early in the project it was agreed that the critical factor for success would lie in creating a simple workflow methodology allowing research teams to autonomously develop study-related metadata in an efficient manner [[36,37]].

Print page

Data Dictionary Codebook

Collapse all instruments

	#	Variable / Field Name	Field Label<br>*Field Note*	Field Attributes (Field Type, Validation, Choices, Calculations, etc.)
Instrument: **Demographics** (demographics)				Collapse
	1	record_id	RedCap ID	text, Required, Identifier<br>Field Annotation: ln_76435_7
	2	sct_184099003	Date of birth<br>*DD-MM-YYYY*	text (date_dmy)
	3	sct_263495000	Biological sex	radio<br>248153007 | Male<br>248152002 | Female<br>Field Annotation: ln_76689_9
	4	sct_372148003	Ethnic group	radio<br>14045001 | Caucasian<br>18167009 | African<br>315280000 | Asian<br>90027003 | Arabic<br>560516 | Hispanic or Latino<br>26242008 | Other ethnic, mixed origin
	5	sct_105421008	Level of education of the patient	radio

Supplementary Figure 1: Data Dictionary Codebook. The Codebook provides a version of the project's Data Dictionary as a quick reference for viewing the attributes of any given field in the eCRFs of the project.

Supplementary References

1. Willett, D. L. *et al.* SNOMED CT Concept Hierarchies for Sharing Definitions of Clinical Conditions Using Electronic Health Record Data. *Appl. Clin. Inform.* **9**, 667–682 (2018).

2. Højen, A. R., Sundvall, E. & Gøeg, K. R. Methods and Applications for Visualization of SNOMED CT Concept Sets. *Appl. Clin. Inform.* **5**, 127–152 (2014).

3. Bietenbeck, A. & Streichert, T. Preparing Laboratories for Interconnected Health Care. *Diagn. Basel Switz.* **11**, 1487 (2021).

4. GA4GH (2021). Welcome to the documentation for the phenopacket-schema. URL: https://phenopacket-schema.readthedocs.io/en/latest/index.html. Accessed: 08/17/21 - Cerca con Google.

5. Hollingworth, S. & Kairuz, T. Measuring Medicine Use: Applying ATC/DDD Methodology to Real-World Data. *Pharmacy* **9**, 60 (2021).

6. Antimicrobial consumption - Annual Epidemiological Report for 2019. *European Centre for Disease Prevention and Control* https://www.ecdc.europa.eu/en/publications-data/surveillance-antimicrobial-consumption-europe-2019 (2020).

7. Dolman, L. *et al.* ClinGen advancing genomic data-sharing standards as a GA4GH driver project. *Hum. Mutat.* **39**, 1686–1689 (2018).

8. Contreras, J. L. & Knoppers, B. M. The Genomic Commons. *Annu. Rev. Genomics Hum. Genet.* **19**, 429–453 (2018).

9. de Coronado, S. *et al.* The NCI Thesaurus quality assurance life cycle. *J. Biomed. Inform.* **42**, 530–539 (2009).

10. von Rhein, C. *et al.* Comparison of potency assays to assess SARS-CoV-2 neutralizing antibody capacity in COVID-19 convalescent plasma. *J. Virol. Methods* **288**, 114031 (2021).

11. Bégin, P. *et al.* Convalescent plasma for hospitalized patients with COVID-19: an open-label, randomized controlled trial. *Nat. Med.* 1–13 (2021) doi:10.1038/s41591-021-01488-2.

12. Ashford, P. & Delgado, M. ISBT 128 Standard for Coding Medical Products of Human Origin. *Transfus. Med. Hemotherapy Off. Organ Dtsch. Ges. Transfusionsmedizin Immunhamatologie* **44**, 386–390 (2017).

13. Covid-19 Research-Dataset - Project Information. https://art-decor.org/art-decor/decor-project--covid19f.

14. Logica Implementation Guide: Covid-19. https://covid-19-ig.logicahealth.org/index.html. Accessed 16 Nov 2020.

15. cocos-Corona Component Standards. http://cocos.team. Accessed 16 Nov 2020.

16. Docherty, A. B. *et al.* Features of 20 133 UK patients in hospital with covid-19 using the ISARIC WHO Clinical Characterisation Protocol: prospective observational cohort study. *BMJ* (2020) doi:10.1136/bmj.m1985.

17. ISARIC. COVID-19 CRF. https://isaric.tghn.org/COVID-19-CRF. Accessed 16 Nov 2020.

18. Kurth, F. *et al.* Studying the pathophysiology of coronavirus disease 2019: a protocol for the Berlin prospective COVID-19 patient cohort (Pa-COVID-19). *Infection* **48**, 619–626 (2020).

19. Jakob, C. E. M. *et al.* First results of the 'Lean European Open Survey on SARS-CoV-2-Infected Patients (LEOSS)'. *Infection* **49**, 63–73 (2021).

20. Design and evaluation of a data anonymization pipeline to promote Open Science on COVID-19 | Scientific Data. https://www.nature.com/articles/s41597-020-00773-y.

21. Sass, J. *et al.* The German Corona Consensus Dataset (GECCO): a standardized dataset for COVID-19 research in university medicine and beyond. *BMC Med Inf. Decis Mak* **20**, (2020).

22. Kay, S., Cangioli, G. & Nusbaum, M. The International Patient Summary Standard and the Extensibility Requirement. *Stud. Health Technol. Inform.* **273**, 54–62 (2020).

23. Lozupone, M. *et al.* Social Frailty in the COVID-19 Pandemic Era. *Front. Psychiatry* **11**, 1168 (2020).

24. M, M. *et al.* Long-COVID: An evolving problem with an extensive impact. *South Afr. Med. J. Suid-Afr. Tydskr. Vir Geneeskd.* **111**, (2020).

25. Akesson, J., Ashworth-Hayes, S., Hahn, R. C., Metcalfe, R. & Rasooly, I. Fatalism, Beliefs, and Behaviors During the COVID-19 Pandemic. (2020) doi:10.3386/w27245.

26. Mahase, E. Covid-19: What do we know about 'long covid'? *BMJ* **370**, m2815 (2020).

27. Castagnoli, R. *et al.* Severe Acute Respiratory Syndrome Coronavirus 2 (SARS-CoV-2) Infection in Children and Adolescents: A Systematic Review. *JAMA Pediatr.* **174**, 882–889 (2020).

28. Dashraath, P. *et al.* Coronavirus disease 2019 (COVID-19) pandemic and pregnancy. *Am. J. Obstet. Gynecol.* **222**, 521–531 (2020).

29. Bhuiyan, M. U. *et al.* Epidemiology of COVID-19 infection in young children under five years: A systematic review and meta-analysis. *Vaccine* **39**, 667–677 (2021).

30. McMichael, T. M. *et al.* Epidemiology of Covid-19 in a Long-Term Care Facility in King County, Washington. *N. Engl. J. Med.* **382**, 2005–2011 (2020).

31. Pereira, M. R. *et al.* COVID-19 in solid organ transplant recipients: Initial report from the US epicenter. *Am. J. Transplant. Off. J. Am. Soc. Transplant. Am. Soc. Transpl. Surg.* **20**, 1800–1808 (2020).

32. Lloyd-Sherlock, P. G. *et al.* WHO must prioritise the needs of older people in its response to the covid-19 pandemic. *BMJ* **368**, m1164 (2020).

33. Fishman, J. A. & Grossi, P. A. Novel Coronavirus-19 (COVID-19) in the immunocompromised transplant recipient: #Flatteningthecurve. *Am. J. Transplant. Off. J. Am. Soc. Transplant. Am. Soc. Transpl. Surg.* **20**, 1765–1767 (2020).

34. Harris, P. A. *et al.* Research electronic data capture (REDCap)--a metadata-driven methodology and workflow process for providing translational research informatics support. *J. Biomed. Inform.* **42**, 377–381 (2009).

35. Harris, P. A. *et al.* The REDCap consortium: Building an international community of software platform partners. *J. Biomed. Inform.* **95**, 103208 (2019).

36. Nadkarni, P. M. & Cheung, K.-H. SQLGEN: A framework for rapid client-server database application development. *Comput. Biomed. Res.* **28**, 479–499 (1995).

37. Fraternali, P. & Paolini, P. Model-Driven Development of Web Applications: The Autoweb System. *ACM Trans. Inf. Syst.* **28**, 60.
