# Supplementary material for: Harmonization and standardization of data for a pan-European cohort on SARS- CoV-2 pandemic
Source: NPJ Digit Med. 2022 Jun 14;5:75. doi: 10.1038/s41746-022-00620-x (PMC9198067; doi:10.1038/s41746-022-00620-x)
Supplement: Supplementary file 2 — Reporting Summary [file 41746_2022_620_MOESM2_ESM.pdf]

nature portfolio

Corresponding author(s): Eugenia Rinaldi

Last updated by author(s): 20.04.2022

Reporting Summary

Nature Portfolio wishes to improve the reproducibility of the work that we publish. This form provides structure for consistency and transparency in reporting. For further information on Nature Portfolio policies, see our Editorial Policies and the Editorial Policy Checklist.

Statistics

For all statistical analyses, confirm that the following items are present in the figure legend, table legend, main text, or Methods section.

n/a	Confirmed	
☒	☐	The exact sample size (n) for each experimental group/condition, given as a discrete number and unit of measurement
☒	☐	A statement on whether measurements were taken from distinct samples or whether the same sample was measured repeatedly
☒	☐	The statistical test(s) used AND whether they are one- or two-sided<br>*Only common tests should be described solely by name; describe more complex techniques in the Methods section.*
☒	☐	A description of all covariates tested
☒	☐	A description of any assumptions or corrections, such as tests of normality and adjustment for multiple comparisons
☒	☐	A full description of the statistical parameters including central tendency (e.g. means) or other basic estimates (e.g. regression coefficient) AND variation (e.g. standard deviation) or associated estimates of uncertainty (e.g. confidence intervals)
☒	☐	For null hypothesis testing, the test statistic (e.g. F, t, r) with confidence intervals, effect sizes, degrees of freedom and P value noted<br>*Give P values as exact values whenever suitable.*
☒	☐	For Bayesian analysis, information on the choice of priors and Markov chain Monte Carlo settings
☒	☐	For hierarchical and complex designs, identification of the appropriate level for tests and full reporting of outcomes
☒	☐	Estimates of effect sizes (e.g. Cohen's d, Pearson's r), indicating how they were calculated

Our web collection on statistics for biologists contains articles on many of the points above.

Software and code

Policy information about availability of computer code

Data collection	The original data set definitions received from clinical partners were analyzed in LibreOffice Calc Version 7.1.1.2. The core data set was developed in Microsoft Excel 2016. All graphs shown in this publication were created in Microsoft PowerPoint 2016 while tables were built in Microsoft Excel 2016. The version of REDCap® used for the work described was 12.0.1. The standard terminologies used are: SNOMED CT International Edition July 2021, LOINC 2.71 ICD-10. ATC/DDD Index 2021, NCIt Version 21.11.e. To look up codes we used International SNOMED CT Browser Version 2021-7-31, SearchLOINC Version 2.20, ICD-10 online application Version 2019, NCI Term Browser Version 2.19.
Data analysis	Analyses were performed using Microsoft Excel 2016.

For manuscripts utilizing custom algorithms or software that are central to the research but not yet described in published literature, software must be made available to editors and reviewers. We strongly encourage code deposition in a community repository (e.g. GitHub). See the Nature Portfolio guidelines for submitting code & software for further information.

Data

Policy information about availability of data

All manuscripts must include a data availability statement. This statement should provide the following information, where applicable:

- Accession codes, unique identifiers, or web links for publicly available datasets
- A description of any restrictions on data availability
- For clinical datasets or third party data, please ensure that the statement adheres to our policy

The metadata definitions for the three data sets are publicly available on the standard-enabling platform ART-DÉCOR (https://art-decor.org/art-decor/decor-project--orch-) . The data sets in ART DÉCOR receive periodic updates to reflect possible changes in the CRFs, consequently, they might differ from the ones we

refer to in the present publication. The Data Dictionaries that were created are stored in a repository online (available at https://cloud.orchestra-cohort.eu/s/HeycD4xY7TACxLX. Access to the files can be granted upon reasonable request.

Field-specific reporting

Please select the one below that is the best fit for your research. If you are not sure, read the appropriate sections before making your selection.

☒ Life sciences ☐ Behavioural & social sciences ☐ Ecological, evolutionary & environmental sciences

For a reference copy of the document with all sections, see nature.com/documents/nr-reporting-summary-flat.pdf

Life sciences study design

All studies must disclose on these points even when the disclosure is negative.

Sample size	We have analyzed metadata for 2558 variables and their related answer lists when available
Data exclusions	No data were excluded
Replication	Variables and their meanings were discussed within the working group before assigning international standard codes. An internal quality check was performed to verify the selected codes
Randomization	Within the eCRFs and respective data dictionaries, variables were divided into defined categories by the clinical study investigators
Blinding	Variables were divided into categories by subject matter experts (clinical study investigator)

Reporting for specific materials, systems and methods

We require information from authors about some types of materials, experimental systems and methods used in many studies. Here, indicate whether each material, system or method listed is relevant to your study. If you are not sure if a list item applies to your research, read the appropriate section before selecting a response.

Materials & experimental systems

n/a	Involved in the study	
☒	☐	Antibodies
☒	☐	Eukaryotic cell lines
☒	☐	Palaeontology and archaeology
☒	☐	Animals and other organisms
☒	☐	Human research participants
☒	☐	Clinical data
☒	☐	Dual use research of concern

Methods

n/a	Involved in the study	
☒	☐	ChIP-seq
☒	☐	Flow cytometry
☒	☐	MRI-based neuroimaging
